# HCC or Something Else? Frequency of Various Benign and Malignant Etiologies in Cirrhotic Patients with Newly Detected Focal Liver Lesions in Relation to Different Clinical and Sonographic Parameters

**DOI:** 10.3390/diagnostics12092079

**Published:** 2022-08-28

**Authors:** Amjad Alhyari, Christian Görg, Raed Alakhras, Christoph Frank Dietrich, Corrina Trenker, Ehsan Safai Zadeh

**Affiliations:** 1 Gastroenterology, Endocrinology, Metabolism and Clinical Infectiology, University Hospital Giessen and Marburg, Philipp University of Marburg, Baldingerstraße, 35033 Marburg, Germany; 2 Interdisciplinary Centre of Ultrasound Diagnostics, University Hospital Giessen and Marburg, Philipp University of Marburg, Baldingerstraße, 35033 Marburg, Germany; 3 Department Allgemeine Innere Medizin (DAIM), Kliniken Hirslanden Bern, Beau Site, Salem und Permanence, 3013 Bern, Switzerland; 4 Haematology, Oncology and Immunology, University Hospital Giessen and Marburg, Philipps University Marburg, Baldingerstraße, 35033 Marburg, Germany

**Keywords:** liver cirrhosis, HCC, ultrasound, liver lesion, HCC screening

## Abstract

Background and Aims: To investigate the frequency of different benign and malignant focal liver lesions (FLLs) in relation to clinical and sonographic features among patients with liver cirrhosis (LC) and newly detected FLLs. Methods: This study was a retrospective analysis of 225 cirrhotic patients with newly detected FLLs who underwent hepatic ultrasound (US) examinations at our university hospital from 2011 to 2022. The diagnosis of FLLs was based on histology and/or consensus radiological criteria, in accordance with the current diagnostic guidelines. The FLLs were classified into benign (bFLLs) or malignant (mFLLs) lesions and the latter group was subclassified into HCC and non-HCC mFLLs. The frequency, clinical parameters, and sonographic features of the different groups were examined and compared. Results: Of the 225 FLLs, 154 (68.4%) were mFLLs and 71 (31.6%) bFLLs. HCC was the most frequent subcategory of FLLs (132; 58.7%). There were (22; 9.8%) non-HCC mFLLs with 11 (4.9%) metastases and 11 (4.9%) non-HCC primary liver tumors. Regenerative nodules (RNs) were the most frequent form of bFLLs (25; 11.1%), followed by simple cysts (22; 9.8%) and hemangiomas (14; 6.2%). The other bFLLs (10; 14.1%) were fat deposition/sparing (5), hematomas (2), abscesses (2), and echinococcal cysts (1). The distribution of bFLLs and HCC and non-HCC mFLLs varied significantly according to the clinical scenarios. HCC mFLLs were more frequent in males (*p* = 0.001), in those with no history of active non-hepatic primary malignant disease (NHPMD) (*p* < 0.001), in those with a hepatitis B or C etiology of LC (*p* = 0.002), when located in the right lobe (*p* = 0.008), and when portal vein thrombosis was present (*p* = 0.03). Conclusion: In cirrhotic patients with newly detected FLLs, the non-HCC etiology was more frequently diagnosed in lesions that were located in the left lobe, in females, and in patients with a history of active NHPMD. Thus, the lower frequency of HCC in the abovementioned groups demonstrated that a cautious implementation of the current consensus radiological criteria would be required for these groups, particularly in patients with an active NHPMD, given the fact that the consensus criteria were not validated in these populations. A more active diagnostic approach may ultimately be needed for these patients. Large prospective studies are needed to validate these findings.

## 1. Introduction

Globally, around 1.5 billion individuals have chronic liver disease [1]. In Europe, the prevalence of liver cirrhosis (LC) has been estimated to be 833/100,000, with an incidence of 26/100,000 per year [1,2,3]. Due to the metabolic syndrome epidemic and the increase in alcohol misuse, the prevalence of LC is increasing despite the wide utilization of the hepatitis B virus (HBV) vaccination and the availability of effective therapy for the hepatitis C virus (HCV) [1].

Hepatocellular carcinoma (HCC) is the fifth most common malignancy and the second leading cause of cancer-related deaths worldwide [4] and its incidence and mortality rates are growing rapidly [5,6]. The annual incidence of HCC among patients with LC, which is by far the strongest risk factor for HCC development, is currently 1–6% [5].

In addition to cirrhosis-related liver lesions (which include regenerative nodules (RNs), low- and high-grade dysplastic nodules, and HCC), many other benign lesions, pseudo-lesions, and non-HCC malignancies can occur in the cirrhotic liver [7,8]. The accurate diagnosis of FLLs in this vulnerable group of patients is imperative.

Imaging plays a pivotal role in the detection and characterization of FLLs. The liver imaging reporting and data system (LI-RADS) is used by radiologists to categorize liver lesions in cirrhotic patients using magnetic resonance imaging (MRI) or computer tomography (CT) in a standardized manner. The ordinal categories range from LR-1 (definitely benign) to LR-5 (definitely HCC), with the additional categories of LR-M for malignancies other than HCC and LR-TIV for tumors in veins [7,9,10,11,12,13]. In a cirrhotic liver, lesions ≥ 1 cm in diameter that show an arterial phase hyperenhancement with delayed washout on multiphasic CT or dynamic enhanced MRI scans (the so called “radiological hallmark of HCC” or LR-5) can be adequately diagnosed as HCC without the need for histology [4,5].

Unlike healthy livers, metastases to cirrhotic livers have been reported to be uncommon in autopsy studies [14,15,16]; however, limited studies have investigated this observation of the relative “immunity” of cirrhotic livers to metastases among living subjects [17,18].

The implementation of ultrasound (US) as the primary screening method for HCC in LC leads to timely detection and improvements in the chances of survival [6]. Additionally, the use of contrast enhanced ultrasound (CEUS) can improve the detection and characterization of different benign and malignant FLLs, including HCC [19], although the evidence for its diagnostic accuracy is considered to be moderate by the European Association for the Study of the Liver (EASL) [4].

The aim of this study was to describe the frequency of different benign (bFLLs) and malignant (mFLLs) focal liver lesions in patients with cirrhosis in relation to clinical and US characteristics.

## 2. Patients and Methods

This study was carried out at a tertiary healthcare center (University Hospital of Marburg) and included 246 consecutive cirrhotic patients with newly diagnosed FLLs between February 2011 to January 2022. The inclusion criteria were: (1) new FLLs in cirrhotic livers that were detected using B-mode US (B-US); (2) a diagnostic confirmation of the FLLs by means of histology, consensus diagnostic criteria on contrast enhanced imaging or both. The diagnosis of simple cysts was confirmed using B-US as the method of choice and the most accurate modality [20]. The clinical, imaging, and pathological data were retrieved and retrospectively analyzed. The study was approved by the local ethics committee (protocol code: RS 22/14) and was conducted in accordance with the amended Declaration of Helsinki. Informed consent was obtained from each patient for the ultrasound examinations.

**Figure 1:**
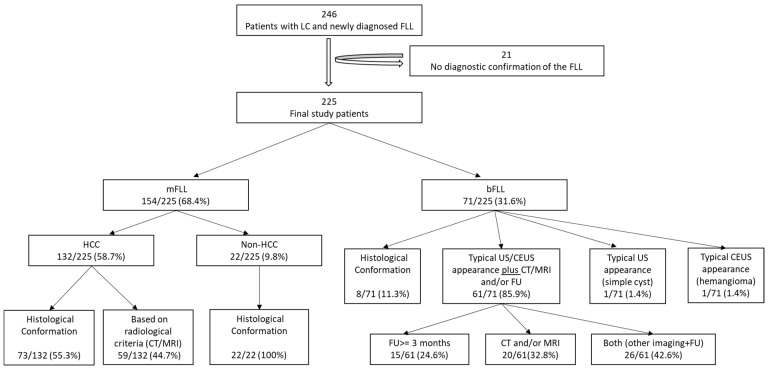
The exclusion and diagnostic confirmation diagram. LC, liver cirrhosis; FLL, focal liver lesion; mFLL, malignant focal liver lesion; bFLL, benign focal liver lesion; HCC, hepatocellular carcinoma; CT, computer tomography; MRI, magnetic resonance imaging; US, ultrasound; CEUS, contrast enhanced ultrasound; FU, follow-up.

In total, 21 of the initial 246 patients (8.5%) were excluded due to the absence of a diagnostic reference. Finally, 225 patients were included in the study analysis. The exclusion diagram is shown in Figure 1. An overview of the final diagnoses of all FLLs is shown in Table 1.

### 2.1. Demographic and Clinical Characteristics of Study Participants

Of the 225 study participants, 173 (76.9%) were male and 52 (23.1%) were female. The mean age of the patients at the time of the US examinations was 65 ± 10 years (range: 18–89 years). All patients had definite sonographic or radiological evidence of liver cirrhosis and the LC was additionally confirmed histologically in 111/225 (49.3%) of the patients. The most common etiology of LC was alcohol (55.1%). Table 2 shows the detailed distribution of LC etiologies among the study participants. Ascites was present in 91 patients (40.4%) and splenomegaly was present in 108 patients (48.6%) (three of those patients (1.3%) were post-splenectomy). The mean model of end-stage liver disease with sodium (MELD-Na) score at the time of the diagnoses of the FLLs was 12.7 ± 6.4 (range: 6–36): 129 of the patients (57.3%) were Child–Pugh–Turcotte (CTP) stage A, 79 (35.1%) were CTP stage B, and 17 (7.6%) were CTP stage C. The mean albumin and platelet counts were 33.0 ± 7.1 g/L and 149.4 ± 87.6 × 10^3^/μL, respectively.

### 2.2. History of Active Non-Hepatic Malignancy

In total, 21 patients (9.3%) had a history of active non-hepatic primary malignant disease (NHPMD) at the time of the first detection of an FLL, including 3 (14.3%) with hematological malignancies, 3 (14.3%) with neuroendocrine tumors, 3 (14.3%) with colorectal carcinomas, 3 (14.3%) with cancer of unknown primary (CUP), 2 (9.5%) with esophageal cancer, 2 (9.5%) cases of gastric cancer, and 1 (4.8%) with each of gallbladder, urinary tract, ovary, lung, and tongue cancer.

### 2.3. Indications to Perform US

The US examinations were carried out as routine HCC screening for 89 of the patients (39.6%). The US examinations were performed to further characterize FLLs that were initially detected on CT/MRI scans in 16 patients (7.1%), to monitor new-onset ascites in 28 patients (12.4%), and to investigate abdominal pain in 30 patients (13.3%). For 62 of the patients (27.6%), the FLLs were incidental findings during US examinations that were performed for other indications.

### 2.4. US and CEUS Examinations

The US examinations were performed using an ACUSON Sequoia 512 GI ultrasound machine (Siemens, Erlangen, Germany) with a 4C1 curved array transducer and a frequency of 4 MHz was used for the B-US investigations. The CEUS examinations were performed using the same transducer in the 1.5 MHz contrast-specific mode, according to the guidelines from the European Federation of Societies for Ultrasound in Medicine and Biology (EFSUMB) [21]. The interpretation of the CEUS images was also performed according to the EFSUMB guidelines [21]. All B-mode US and CEUS examinations and interpretations, as well as all US-guided biopsies, were conducted by a qualified investigator (Level 3 from the German Society of Ultrasound in Medicine (DEGUM)) with more than 35 years of ultrasound experience (C.G., internal medicine) [22]. For the evaluation of the liver lesions after B-US, a CEUS examination was performed on 180/225 (80.0%) patients.

### 2.5. Cross-Sectional Imaging

Cross-sectional imaging was performed for 205/225 patients (91.1%): CT was performed for 92 patients (40.1%), MRI was performed for 39 patients (17.3%), and both were performed for 74 patients (32.9%). Cross-sectional imaging was available for 152/154 (98.7%) malignant FLLs (mFLLs) and 53/71 (74.6%) benign FLLs (bFLLs).

The consensus diagnostic criteria from the 2018 EASL guidelines were utilized for the diagnosis of HCC, namely lesions ≥ 1 cm in diameter in a cirrhotic liver that showed an arterial phase hyperenhancement (APHE) with washout in the portal venous or delayed phases on multiphasic CT or dynamic MRI scans [19].

### 2.6. Histological Specimens

Histological diagnosis was available for 103/225 (45.8%) FLLs, including 87/103 (84.5%) US-guided transabdominal liver biopsies, 13/103 (12.6%) surgical specimens, and 3/103 (2.9%) autopsy specimens. All tissue specimens were examined by two pathologists, who were experienced in gastrointestinal diseases, at a tertiary university hospital.

### 2.7. Diagnostic Confirmation of FLLs

**Figure 2:**
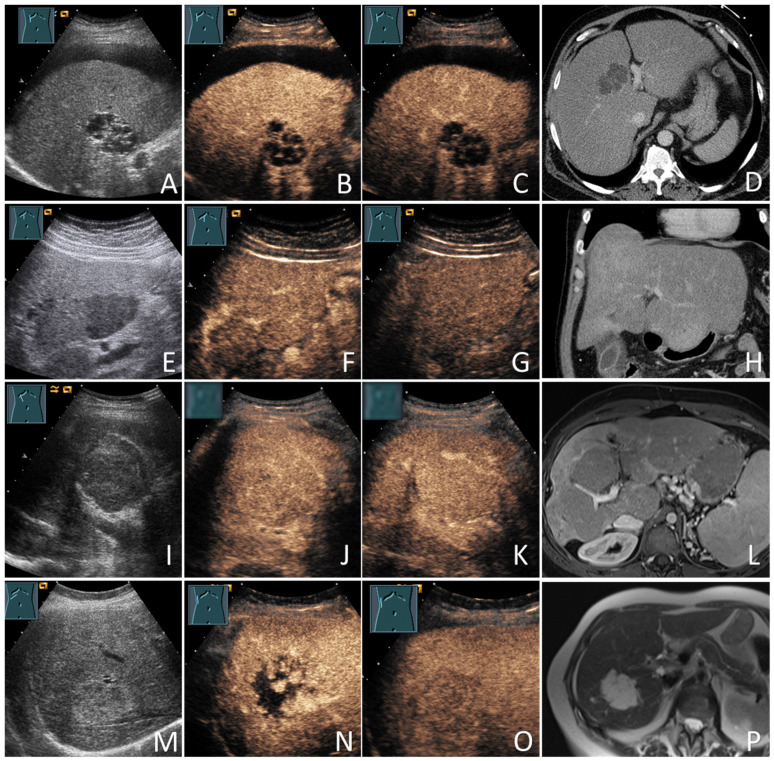
Benign focal liver lesions (bFLLs) in patients with liver cirrhosis (LC). Liver cyst (**A–D**): hypoechoic FLL with multiple septa on grey-scale ultrasound (**A**); hypoechoic FLL with arterial phase isoenhancement of the septa (**B**); hypoechoic FLL without washout in the delayed venous phase (**C**); the corresponding CT of the FLL (courtesy of Prof. Dr. Andreas H. Mahnken, Department of Radiology, University Hospital Marburg) (**D**). Focal fat sparing (**E**–**H**): hypoechoic FLL on grey-scale ultrasound (**E**); hypoechoic FLL with isoenhancement on the arterial phase (**F**); hypoechoic FLL with isoenhancement on the delayed venous phase (**G**); the corresponding CT of the FLL (courtesy of Prof. Dr. Andreas H. Mahnken, Department of Radiology, University Hospital Marburg) (**H**). Regenerative nodules (**I–L**): large central isoechoic lesion on grey-scale ultrasound (**I**); large central isoechoic lesion with isoenhancement on the arterial phase (**J**); large central isoechoic lesion with isoenhancement on the delayed venous phase (**K**); the corresponding MRI of the FLL (courtesy of Prof. Dr. Andreas H. Mahnken, Department of Radiology, University Hospital Marburg) (**L**). Hemangiomas (**M**–**P**): large central echogenic lesion on grey-scale ultrasound (**M**); large central echogenic lesion with nodular enhancement on the arterial phase (**N**); large central echogenic lesion with isoenhancement on the venous phase (**O**); the corresponding MRI of the FLL (courtesy of Prof. Dr. Andreas H. Mahnken, Department of Radiology, University Hospital Marburg) (**P**). The lesions remained stable over a follow-up period of over 92 months.

**Figure 3:**
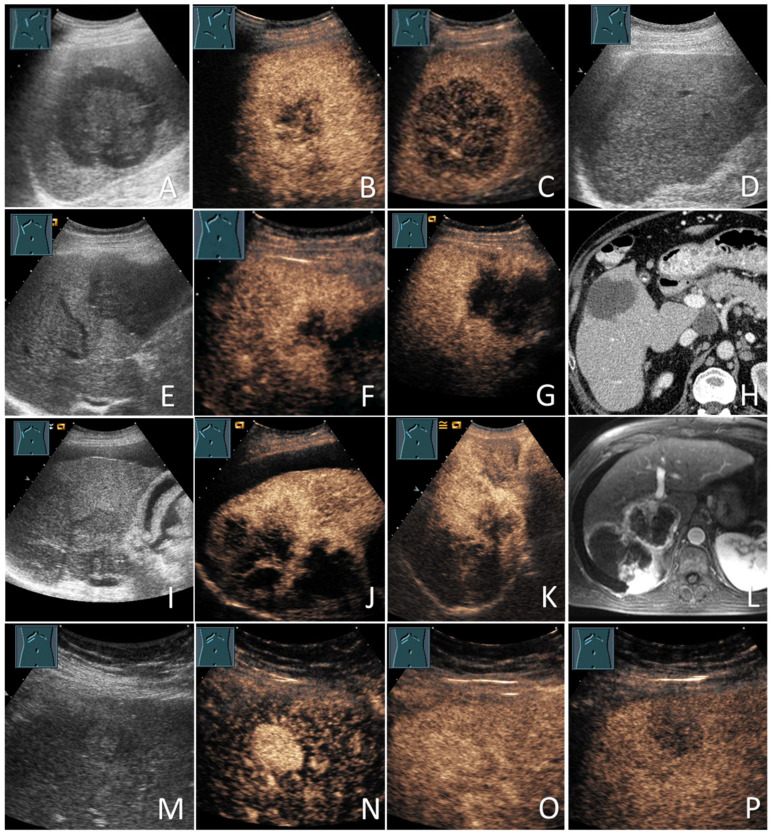
Malignant focal liver lesions (mFLLs) in patients with liver cirrhosis (LC). Lymphoma (the biopsy showed diffused large B-cell lymphoma) (**A–D**): large hypoechoic FLL on grey-scale ultrasound (**A**); large hypoechoic FLL with inhomogeneous enhancement on the arterial phase (**B**); large hypoechoic FLL with marked washout in the delayed venous phase (**C**); the sonographic follow-up image with the near total resolution of the lesions after chemotherapy (**D**). Metastases (the biopsy showed an adenocarcinoma and the primary tumor was unknown (CUP)) (**E–H**): hypoechoic FLL on grey-scale ultrasound (**E**); hypoechoic FLL with inhomogeneous peripheral isoenhancement on the arterial phase (**F**); hypoechoic FLL with early washout within 2 min (**G**); the corresponding CT of the FLL (courtesy of Prof. Dr. Andreas H. Mahnken, Department of Radiology, University Hospital Marburg) (**H**). Cholangiocarcinoma (the biopsy showed an intrahepatic cholangiocarcinoma) (**I–L**): large lobulated hypoechoic lesion on grey-scale ultrasound (**I**); large lobulated hypoechoic lesion with inhomogeneous peripheral enhancement after 20 s (**J**) and 60 s (**K**); the corresponding MRI of the FLL (courtesy of Prof. Dr. Andreas H. Mahnken, Department of Radiology, University Hospital Marburg) (**L**). Hepatocellular carcinomas (the lesions were proved to be moderately differentiated HCC on the histology) (**M–P**): small echogenic lesion on grey-scale ultrasound (**M**); small echogenic lesion with an early arterial phase hyperenhancement after 12 s (**N**) small echogenic lesion with isoenhancement after 40 s (**O**); small echogenic lesion washout after 3 min (**P**).

In the cases of HCC, the diagnoses were based on histology in 73/132 patients (55.3%) and the diagnostic radiological criteria in 59/132 patients (44.7%). All non-HCC mFLLs were diagnosed based on histology (22/22; 100%). Of the 71 bFLLs, 8 (11.3%) were diagnosed using histology and the remaining 63 (88.7%) were diagnosed using characteristic CEUS (e.g., hemangiomas) or sonographic appearance (e.g., simple cysts) [20,21]. The benign nature of these FLLs was further supported by cross-sectional imaging and/or sonographic FUs in 61/71 patients (85.9%), with a mean FU duration of 35.6 ± 25.5 months. Figure 1 provides information about the verification of the diagnoses of all study participants. Figure 2 and Figure 3 show some examples of the benign and malignant FLLs that were included in this study.

### 2.8. Statistical Analysis

Continuous variables were expressed as the mean values ± standard deviations (SDs). The statistical evaluation was performed on the categorical variables using the Chi-squared and Fisher’s exact tests and the continuous variables using the Mann–Whitney U and Kruskal–Wallis tests. A *p*-value of <0.05 was defined as significant. The statistical analyses were performed using Excel (Microsoft 365 MSO; Microsoft Corporation, Redmond, WA, USA) and SPSS version 26.0 statistics software (IBM, Armonk, NY, USA).

## 3. Results

### 3.1. Final Etiologies of the FLLs

There were 71/225 (31.6%) bFLLs and 154/225 (68.4%) mFLLs. The most common form of bFLLs was RNs (25/225; 11.1%), followed by simple cysts (22/225; 9.8%) and hemangiomas (14/225; 6.2%). On the other hand, out of all of the FLLs, HCC was the most frequent etiology (132/225; 58.7%), representing 85.7% or 132/154 of all mFLLs. Non-HCC mFLLs were present in 22/225 patients (9.8%), including 11/225 (4.9%) metastases and 11/225 (4.9%) primary non-HCC liver tumors. Of the latter etiology, intrahepatic cholangiocarcinoma (ICC) was the most common form (9/225; 4.0%) (Table 1).

### 3.2. Clinical Features

**Figure 4:**
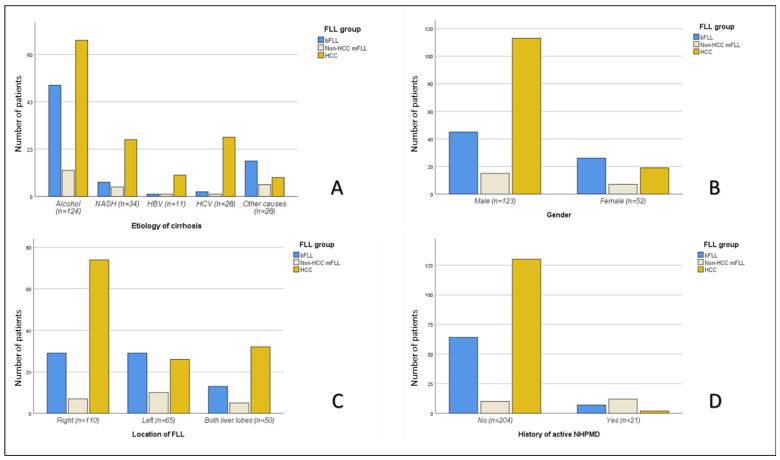
The differences in the etiological distribution of the three major FLL groups (bFLLs and HCC and non-HCC mFLLs) among the 225 study subjects, according to the etiology of cirrhosis (**A**), gender (**B**), lobar distribution (**C**), and history of active non-hepatic primary malignant disease (NHPMD) (**D**). FLL, focal liver lesion; bFLL, benign focal liver lesion; mFLL, malignant focal liver lesion; HCC, hepatocellular carcinoma.

Malignant FLLs were more frequent in older (*p* < 0.001) and male (*p* = 0.002) subjects. Moreover, HCC was more frequently encountered in males (113/173; 65.3%) compared to females (19/52; 36.5%), with *p =* 0.001 (Figure 4 and Table 3).

The overall frequency of mFLLs did not differ significantly among patients with and without a history of active NHPMD (14/21 or 66.7% vs. 140/204 or 68.6%; *p* > 0.05). However, HCC was significantly less frequent in the group with a history of NHPMD (2/21 or 9.5% vs. 130/204 or 63.7%; *p* < 0.001) (Figure 4).

The overall frequency of mFLLs and HCC did not differ significantly according to the CTP stage or MELD-Na score (*p > 0.05)* (Table 3).

There was a significant association between the frequency of HCC and the etiology of LC (*p =* 0.002), with the highest frequency of FLLs detected in those with HCV-related LC (26/28; 92.9%) and HBV-related LC (10/11; 90.9%) (Figure 4).

### 3.3. Laboratory Parameters

No significant associations were found between albumin level or platelet count and the etiologies of the FLLs.

AFP values were available for 185/225 patients (82.2%), including 120/132 (90.9%) HCC mFLLs, 16/22 (72.7%) non-HCC mFLLs, and 49/71 (69.0%) bFLLs. The mean AFP values (in ng/dl) were significantly higher in patients with HCC mFLLs (2197 ± 9093) compared to those with non-HCC mFLLs (55 ± 173) or bFLLs (5.3 ± 5.8), *p* < 0.001. No significant differences were found between non-HCC mFLLs and bFLLs, *p* > 0.05 (Table 3).

### 3.4. Sonographic Features

The sonographic characteristics of the study participant are presented in Table 3. Among the FLLs, 162/225 (72.0%) were hypoechoic and 63/225 (28.0%) were echogenic, although the frequency of mFLLs did not differ between hypoechoic FLLs (112/162; 69.1%) and echogenic FLLs (42/63; 66.7%). The mean size (in cm) of all of the FLLs was 4.1 ± 3.1: bFLLs = 2.2 ± 1.7, mFLLs = 5.0 ± 3.1, HCC mFLLs = 4.9 ± 3.0, and non-HCC mFLLs = 5.3 ± 3.8. The mean size of the mFLLs was significantly higher than that of the bFLLs (*p* < 0.001). There were no significant correlations between the presence of ascites, the number of FLLs or the presence of splenomegaly with the overall frequency of mFLLs or HCC (*p >* 0.05). FLLs that were located in the right liver lobe (110/225; 48.9%) or both lobes (50/225; 22.2%) were more frequently malignant (81/110; 73.6% and 37/50; 74.0%, respectively) compared to FFLs that were located in the left lobe (65/225; 28.9%), of which 36 (55.4%) were malignant (*p* = 0.03). Moreover, HCC was less frequent in FLLs that were located in the left liver lobe (26/65; 40.0%) compared to those that were located in the right liver lobe (74/111; 67.3%) or both lobes (32/50; 64%) (*p* = 0.008) (Figure 4). Portal vein thrombosis (PVT) was present in 25/225 (11.1%) FLLs, of which 18 (72.0%) were due to tumor invasion (or “living thrombus”) and 7 (28.0%) were due to blood clots. PVT was present in 2/71 (2.8%) bFLLs and 23/154 (14.9%) mFLLs (*p* = 0.006). In the HCC group, PVT was present in 20/132 (15.2%) compared to 5/93 (5.4%) in the non-HCC group (*p =* 0.03).

## 4. Discussion

LC patients are at an increased risk of developing HCC, so they require regular US surveillance. However, lesions other than those caused by HCC can complicate clinical investigations and mandate further noninvasive and/or invasive evaluation. Thus, the correct identification of FLLs is imperative in these vulnerable patients. In this standardized study, we retrospectively assessed the prevalence of different etiologies of newly detected FLLs among patients with LC.

In this study, 31.6% (71/225) of the FLLs were benign and 68.4% (154/225) were malignant. In a study on cirrhotic patients with FLLs that was carried out by Seitz et al., the frequency of malignancy was reported to be 84.0% [17]; however, it is worth mentioning that Seitz excluded patients with simple cysts. In fact, when we excluded simple cysts (*n* = 22) in our study, the prevalence of malignancy was similar (154/203; 75.9%). As is already known, the risk of FLLs being malignant is higher in cirrhotic patients than that in asymptomatic subjects with healthy livers [23] and even those with active systemic malignancies [24,25] (Table 4).

HCC was the most frequent etiology among all of the FLLs (132/225; 58.7%) and accounted for 85.7% (132/154) of all mFLLs. This was slightly lower than the frequency of HCC that was reported by Seitz et al. (76.6% of all FLLs and 91.1% of mFLLs) [17].

The overall frequency of mFLLs was significantly higher in males and older subjects. Both age and gender are well-established risk factors for HCC [26,27]. Moreover, the frequency of mFLLs in cirrhotic males (128/173; 74.0%) was higher than that in females (26/52; 50.0%). This difference between the sexes was even more pronounced for HCC prevalence (113/173 or 65.3% for males vs. 19/52 or 36.5% for females) (Figure 4). Despite the fact that gender disparity in HCC incidence is a well-documented phenomenon with a male to female ratio of 2:8, the exact cause for this disparity remains unknown [27]. Some studies have suggested that it is due to the different sex hormones, with estrogen playing a protective role through the suppression of proinflammatory cytokines, such as IL-6, and testosterone playing a stimulative role via the upregulation of vascular endothelial growth factor [27,28]. Additionally, bFLLs are generally more common in females [29,30].

The overall frequency of mFLLs and HCC did not differ according to CTP stage, MELD-Na score, albumin level or platelet count. As expected, the mean AFP values were significantly higher in patients with HCC compared to those with bFLLs or non-HCC mFLLs.

Regarding the sonographic features, there were no significant differences among the three groups (bFLLs and HCC and non-HCC mFLLs) with regard to ascites, splenomegaly, echogenicity, and the number of FLLs per patient. However, the etiologies of the FLLs varied according to their location in the liver, with right or bilobar distributions having a higher prevalence of malignancy (73.6% and 74.0% vs. 28.9%). Additionally, HCC was more frequently diagnosed in FLLs that were located in the right or both lobes compared to those that were located in the left lobe (67.3% and 64.0% vs. 40.0%). Data on the lobar/segmental distribution of hepatic lesions as a risk factor for malignancy and/or HCC in cirrhotic, as well as non-cirrhotic, patients are scarce. One study reported a higher risk for the microvascular invasion of HCC lesions that were located in the right liver lobe [31]. The size of the mFLLs was significantly larger than that of the bFLLs, although no significant size differences between HCC and non-HCC mFLLs were found. Moreover, PVT was more frequently encountered in mFLLs (*p* = 0.006) and HCC lesions were more frequently accompanied by PVT than non-HCC lesions (*p* = 0.03). PVT is a known poor prognostic factor in HCC [32,33].

There were significant differences in the frequency of HCC among the different etiologies of LC, with the highest frequency found among FLLs that were detected in those with LC due to chronic infection with HCV (26/28; 92.9%) and HBV (10/11; 90.9%) (Figure 4). Patients with LC due to chronic HCV infection have a high risk of HCC, with an annual incidence of up to 10%, and chronic hepatitis B is the leading cause of HCC worldwide. NASH is currently the fastest growing indication for HCC-related liver transplants in the United States [26].

Although the overall frequency of malignancy (mFLLs) did not differ significantly between those with and without active NHPMD (14/21 or 66.7% vs. 140/204 or 68.6%; *p* > 0.05), HCC was significantly less frequent in the group with a history of active NHPMD (2/21 or 9.5% vs. 130/204 or 63.7%; *p* < 0.001) (Figure 4). This was finding was contrary to the HCC frequency in patients with (76.7%) and without (76.6%) a history of NHPMD that was reported by Seitz et al. [17]. In our study, all of the 21 NHPMD cases were active at the time of the FLL detection. It was not reported whether the patients with a “known history of extrahepatic malignancy” in the study by Seitz et al. had active malignancies at the time of FLL diagnosis. Further studies are needed to verify the actual prevalence of HCC among patients with active NHPMD. On the other hand, 11/21 (52.4%) patients with active NHPMD had metastatic FLLs, which was comparable to the results from the study of Seitz et al. (12/30; 40%) [17] and was located in the upper range of the prevalence of 0–39% that has been reported by autopsy studies on patients with cirrhosis and extrahepatic malignancies from 1942 to 2021 [18]. The overall prevalence of metastases among all of the patients with LC in this study was low (11/225; 4.9%), which was similar to the study by Seitz et al. (4.3%) [17] and the autopsy studies (0.6–6.5%) [18]. This prevalence was much lower those that in non-cirrhotic patients with FLLs and patients with synchronous non-hematological [25] or hematological active malignancies [24]. Nevertheless, it is important to keep in mind the possibility of metastatic mFLLs in patients with LC since these lesions can be misdiagnosed as HCC on imaging [34], which can lead to incorrect management (Figure 3A–D). The most frequent primary tumor for hepatic metastases in our study was CUP (3/11; 27.3%), followed by CRC, NET, and lymphoma (2/11 each; 18.2%), and esophageal and gallbladder cancers (1/11 each; 9.1%). While the colorectum is the most common single primary origin for liver metastases in LC, as reported in the literature, most of these metastases were from other locations and mandated wide differential diagnoses [18]. The rarity of metastases in LC could be explained by the “seed and soil hypothesis”, as described by Paget in 1889, in which metastatic “seeds” only thrive within “favorable soil”, such as normal livers. In such cases, the distorted fibrotic microenvironment of a cirrhotic liver offers an “unfavorable soil” for “seeds” to grow and thrive [18]. Another explanation was offered by Ewing in 1928, who suggested that hemodynamic changes are involved in the development and distribution of metastases; so, in the case of LC, the sinusoidal obstruction, increased resistance to or reversal of portal venous flow and/or the presence of portosystemic shunts may preclude the liver from metastatic involvement in many instances [18]. Among the remaining 10 FLLs in patients with a history of active NHPMD in our study, 2 (9.5%) were HCC, 1 (4.8%) was ICC, and 7 (33.3%) were bFLLs, including 2 RNs, 2 hemangiomas, 2 FDS, and 1 simple cyst.

Among the 22 non-HCC mFLLs, primary non-HCC liver tumors were encountered in 11 patients (50%), with an overall frequency of 11/225 (4.9%), including 9 ICC, 1 mixed HCC/ICC [35], and 1 angiosarcoma. Although the overall prevalence of ICC in cirrhosis was low (9/225; 4.0%), it was higher than that reported by Seitz et al. (2.5%) [17]. This could reflect the overall increase in the prevalence of ICC in Germany [36].

Regarding the current LI-RADS criteria, the LR-5 category is intended to have a 100% accuracy for diagnosing HCC in cirrhotic livers; however, the sensitivity of the LR-5 criteria for HCC is moderate and the categories of LR-4 and below do not exclude HCC [7]. Moreover, there has been evidence that a significant percentage of lesions that were initially assigned as LR-M were subsequently proven to be HCC [9]. Additionally, caution is recommended when assigning the LR-5 category to FLLs in patients with known non-hepatic malignancies because the LI-RADS criteria were not validated in such patients [9].

The frequency of bFLLs was 71/225 (31.6%), with RNs being the most common form (25/225; 11.1%), followed by simple cysts (22/225; 9.8%) and hemangiomas (14/225; 6.2%). Fat deposition/sparing (FDS) was present in 5/225 patients (2.2%). The reaming bFLLs included two abscesses (0.9%), two hematomas (0.9%), and one echinococcal cyst (0.4%). In comparison, the frequency of RNs, hemangiomas, and FDS in the study by Seitz et al. (liver cysts were not included) were 5.7%, 2.8%, and 0.3%, respectively [17]. The lower frequency of the above benign lesions could be attributed to the lower overall number of bFLLs in the study by Seitz et al. (42/282; 14.9%) and the better detection of FDS and small hemangiomas by US and CEUS in comparison to CT/MRI [37]. In general, hemangiomas are much less frequently encountered in LC compared to normal livers, which may reflect the regression of such lesions due to fibrotic and necrotic changes in the parenchyma [17,38]. No focal nodular hyperplasia (FNH) or adenomas were encountered in our sample and these benign lesions have been reported to be rarely detected in LC [17]. Table 5 summarizes some of the published studies on the prevalence of different benign and malignant FLLs in patients with LC [9].

Finally, there were some limitations to this study. Firstly, this study was limited by its retrospective nature and by it being a single-center study from a tertiary university hospital with a relatively small number of patients. Secondly, histological confirmation was not available for all FLLs. Nevertheless, all hepatic lesions were diagnosed either by histology or in accordance with the current radiologic consensus criteria. Large prospective studies are needed to address the actual degree of diagnostic concordance/discordance between the different contrast enhanced imaging techniques and pathohistological examinations. These studies should particularly include patients with active NHPMD.

## 5. Conclusions

Even though HCC is the most common form of FLLs in patients with LC and other benign and non-HCC malignant FLLs are less frequently encountered, non-HCC FLLs do occur in cirrhotic livers with a variable frequency, depending on the clinical scenario. Thus, it is imperative to consider these differential diagnoses whenever FLLs are encountered in cirrhotic livers. In this study, the lower frequency of HCC among females, patients with a history of active non-hepatic malignancies, and lesions that were restricted to the left lobe of the liver, alongside the growing concern regarding the moderate sensitivity and overlapping features of some of the LI-RADS categories, signified the need for the cautious implementation of the current diagnostic radiological consensus and could advocate for a more active approach in pursuing histological diagnoses in clinical settings. This would be particularly important for patients with active non-hepatic malignancies given the fact that the LI-RADS system was not validated for this population.

## Figures and Tables

**Table 1:** The final diagnoses of all 225 FLLs.

Group	bFLLs	mFLLs
*n*	71	154
Disease Entity	RNs (25) Simple Cysts (22) Hemangiomas (14) FDS (5) Abscesses (2) Hematomas (2) Echinococcal Cysts (1)	HCC (132)
		Metastases (11)
		CUP (3) CRC (2) NET (2) Lymphoma (2) Gallbladder (1) Esophageal (1)
		ICC (9)
		Mixed HCC/ICC (1)
		Angiosarcomas (1)

FLL, focal liver lesion; bFLL, benign focal liver lesion; mFLL, malignant focal liver lesion; RN, regenerative nodule; FDS, fat deposition/sparing; HCC, hepatocellular carcinoma; CUP, cancer of unknown primary; CRC, colorectal carcinoma; NET, neuroendocrine tumor; ICC, intrahepatic cholangiocarcinoma.

**Table 2:** The distribution of the various etiologies of cirrhosis among the 225 study participants. NASH, non-alcoholic steatohepatitis; HCV, hepatitis C virus; HBV, hepatitis B virus; AIH, autoimmune hepatitis; PSC, primary sclerosing cholangitis; PBC, primary biliary cirrhosis.

Etiology	*n*	Percentage
Alcohol	124	55.1
NASH	34	15.1
HCV	28	12.4
HBV	11	4.9
Hemochromatosis	7	3.1
AIH	4	1.8
PSC	4	1.8
PBC	4	1.8
Other *	2	0.9
Idiopathic	7	3.1

* One case of cardiac cirrhosis and one case of Budd–Chiari syndrome.

**Table 3:** The distribution of the different FLL groups according to the clinical, laboratory, and sonographic characteristics of the 225 study participants.

Group	bFLLs	HCC mFLLs	Non-HCC mFLLs	*p*-Value
*n*	71	132	22	
Age (years)	61 ± 11	67 ± 9	66 ± 6	<0.001
Male (%)	63	86	68	0.001
History of Active NHPMD (%)	7 (9.9)	2 (1.5)	12 (55)	<0.001
CTP Stage: A/B/C (%)	60/30/10	56/36/8	55/45/0	0.45
MELD-Na Score	12.9 ± 6.5	12.6 ± 6.2	12.2 ± 7.4	0.69
Albumin (g/L)	33.5 ± 7.7	33.1 ± 6.9	31.0 ± 6.3	0.31
Platelet Count (×10^3^/μL)	152 ± 95	147 ± 82	155 ± 96	0.98
AFP (ng/dL)	5.3 ± 5.8 *	2197 ± 9093	55 ± 173 ^#^	<0.001
Size of the Lesion (cm)	2.2 ± 1.7	4.9 ± 3.0	5.3 ± 3.8	<0.001
Lobar Location: Right/Left/both (%)	41/41/18	56/20/24	32/45/23	0.008
Ascites: *n* (%)	30 (42)	50 (38)	11 (50)	0.52
Splenomegaly: *n* (%)	38 (54) **	63 (48) ^§^	7 (32) ^$^	0.24
Hypoechoic Echogenicity: *n* (%)	50 (70)	95 (9)	17 (77)	0.82
Portal Vein Thrombosis (%)	2 (2.8)	20 (15.2)	3 (13.6)	0.03

FLL, focal liver lesion; bFLL, benign focal liver lesion; mFLL, malignant focal liver lesion; HCC, hepatocellular carcinoma; NHPMD, non-hepatic primary malignant disease; CTP, Child–Pugh–Turcotte; MELD-Na, model for end-stage liver disease with sodium; AFP, alpha-fetoprotein; g/L, gram per liter; ng/dL, nanogram per deciliter; cm, centimeter; * data available for *n* = 49/71 patients; ^#^ data available for *n* = 16/22 patients; ** data available for *n* = 70/71 patients (*n* = 1 patient post-splenectomy); ^$^ data available for *n* = 21/22 patients (*n* = 1 patient post-splenectomy); ^§^ data available for *n* = 131/132 patients (*n* = 1 patient post-splenectomy).

**Table 4:** The frequency of malignant FLLs, depending on the presence or absence of cirrhosis and non-hepatic primary malignant disease. FLL, focal liver lesion.

Clinical Background	Prevalence of mFLLs (%)	*n*	Year	Author
Incidental detection in asymptomatic patients	0.6	542	2016	Choi et al. [23]
Patients with synchronous hematological malignancies	33.0	61	2013	Heller et al. [24]
Patients with synchronous non-hematological malignancies	59.4	434	2021	Safai Zadeh et al. [25]
Patients with liver cirrhosis	76.6	282	2011	Seitz et al. [17]
Patients with liver cirrhosis	68.4	228	2022	Present Study

**Table 5:** A summary of some of the studies on the prevalence of different benign and malignant FLLs in patients with liver cirrhosis.

Study	*n*	Male (%)	Imaging Modality	Final Diagnosis		
				HCC (%)	Non-HCC mFLLs (%)	bFLLs (%)
Abd Alkhalik Basha et al., 2017 [39]	55	58	CT	34 (61.8)	2 (3.6)	19 (34.5)
Allen et al., 2018 [40]	57	NR	MRI	36 (63.2)	NR	21 (36.8)
An et al., 2017 [41]	225	77	MRI	218 (96.9)	7 (3.1)	0
Burke et al., 2016 [42]	30	NR	MRI	20 (66.7)	NR	10 (33.3)
Cerny et al., 2018 [43]	275	74	MRI	113 (41.1)	10 (3.6)	152 (55.3)
Cha et al., 2017 [44]	445	72	CT + MRI	397 (89.2)	31 (7.0)	17 (3.8)
Channual et al., 2014 [45]	131	NR	MRI	116 (88.5)	NR	NR
Choi et al., 2016 [46]	379	84	MRI	327 (86.3)	9 (2.4)	43 (11.3)
Fraum et al., 2018 [47]	220	74	CT + MRI	136 (61.8)	42 (19.1)	42 (19.1)
Horvat et al., 2018 [48]	102	54	MRI	51 (50.0)	51 (50.0)	0
Joo et al., 2016 [49]	106	79	MRI	71 (67.0)	35 (33.0)	0
Kim et al., 2017 [50]	112	69	CT + MRI	75 (67.0)	0	37 (33.0)
Kim et al., 2018 [51]	202	83	MRI	129 (63.9)	6 (3.0)	67 (33.1)
Lee et al., 2018 [52]	133	75	MRI	107 (80.4)	3 (2.3)	23 (17.3)
Liu et al., 2018 [53]	297	86	CT + MRI	178 (59.9)	13 (4.4)	106 (35.6)
Qi et al., 2016 [54]	192	NR	MRI	138 (71.9)	0	54 (28.1)
Ronot et al., 2017 [55]	595	81	CT + MRI	341 (57.3)	8 (1.3)	NR
Seitz et al., 2011 [17]	282	80	CT + MRI + US + CEUS	216 (76.6)	21 (7.4)	42 (14.9)
Present Study	225	77	CT + MRI + US + CEUS	132 (58.7)	22 (9.8)	71 (31.6)

FLL, focal liver lesion; bFLL, benign focal liver lesion; mFLL, focal liver lesion; HCC, hepatocellular carcinoma; CT, computer tomography; MRI, magnetic resonance imaging; NR, not reported; US, ultrasound; CEUS, contrast enhanced ultrasound; FU, follow-up.

## Data Availability

The data presented in this study are available on request from the corresponding author.

## Abbreviations

AFP	Alpha-fetoprotein
AIH	Autoimmune hepatitis
APHE	Arterial phase hyperenhancement
bFLLs	Benign focal liver lesions
B-US	B-mode ultrasound
CEUS	Contrast enhanced ultrasound
CRC	Colorectal carcinoma
CT	Computer tomography
CTP	Child–Pugh–Turcotte
CUP	Cancer of unknown primary
DEGUM	German Society of Ultrasound in Medicine
EASL	European Association for the Study of the Liver
EFSUMB	European Federation of Societies for Ultrasound in Medicine and Biology
FDS	Fat deposition/sparing
FLLs	Focal liver lesions
FU	Follow-up
HBV	Hepatitis B virus
HCC	Hepatocellular carcinoma
HCV	Hepatitis C virus
ICC	Intrahepatic cholangiocarcinoma
LC	Liver cirrhosis
LI-RADS	Liver imaging reporting and data system
MELD-Na	Model of end-stage liver disease with sodium
mFLLs	Malignant focal liver lesions
MRI	Magnetic resonance imaging
NASH	Non-alcoholic steatohepatitis
NET	Neuroendocrine tumor
NHPMD	Non-hepatic primary malignant disease
PBC	Primary biliary cirrhosis
PSC	Primary sclerosing cholangitis
PVT	Portal vein thrombosis
RN	Regenerative nodule
SD	Standard deviation
TIV	Tumor in a vein
US	Ultrasound
